# A Biohybrid Setup for Coupling Biological and Neuromorphic Neural Networks

**DOI:** 10.3389/fnins.2019.00432

**Published:** 2019-05-08

**Authors:** Hanna Keren, Johannes Partzsch, Shimon Marom, Christian G. Mayr

**Affiliations:** ^1^Department of Physiology, Biophysics and Systems Biology, Ruth and Bruce Rappaport Faculty of Medicine, Technion – Israel Institute of Technology, Haifa, Israel; ^2^Network Biology Research Laboratory, Faculty of Electrical Engineering, Technion – Israel Institute of Technology, Haifa, Israel; ^3^Institute of Circuits and Systems, Faculty of Electrical and Computer Engineering, School of Engineering Sciences, Dresden University of Technology, Dresden, Germany

**Keywords:** neural engineering, brain-machine interfacing, neural networks, neuromorphic networks, neural coupling

## Abstract

Developing technologies for coupling neural activity and artificial neural components, is key for advancing neural interfaces and neuroprosthetics. We present a biohybrid experimental setting, where the activity of a biological neural network is coupled to a biomimetic hardware network. The implementation of the hardware network (denoted NeuroSoC) exhibits complex dynamics with a multiplicity of time-scales, emulating 2880 neurons and 12.7 M synapses, designed on a VLSI chip. This network is coupled to a neural network *in vitro*, where the activities of both the biological and the hardware networks can be recorded, processed, and integrated bidirectionally in real-time. This experimental setup enables an adjustable and well-monitored coupling, while providing access to key functional features of neural networks. We demonstrate the feasibility to functionally couple the two networks and to implement control circuits to modify the biohybrid activity. Overall, we provide an experimental model for neuromorphic-neural interfaces, hopefully to advance the capability to interface with neural activity, and with its irregularities in pathology.

## Introduction

Developing interfaces between brain activity and electrical circuits could bring new perspectives for basic research and medical applications, as therapeutic brain stimulation or neuroprosthetics. The development of such interfaces involves multiple challenges and expertise, spanning the fields of neurobiology, electrophysiology, bioengineering, computational neuroscience, and neuromorphic electrical engineering. Among the challenges are the different organizational levels of the neural systems involved, from *in vitro* to the whole brain; the different methodologies for stimulating and recording neural activity; various signal processing tools for detecting neural activity; biomimetic designs in hardware; and a variety of neural networks modeling approaches (Broccard et al., 2017; Chiolerio et al., 2017). The attempts which have been made to implement such interfaces show great promise (O’Doherty et al., 2011; Capogrosso et al., 2016; Joucla et al., 2016), and therefore, immense efforts are invested in tackling this challenge.

For example, novel neurotherapeutic devices [see Greenwald et al. (2016) for review], use neural stimulation to help with epilepsy (Vagus Nerve Stimulation Study Group, 1995; Fisher and Velasco, 2014), chronic pain (Kumar et al., 2007), and rehabilitation following spinal cord injury (Harkema et al., 2011; Angeli et al., 2014). Other studies use bidirectional stimulation to develop motoric feedback interfaces (O’Doherty et al., 2011; Vato et al., 2012), or to reinstate the input output relations of brain regions (Berger et al., 2012). These methods often modify the stimulation parameters based on response biomarkers, at the time of initiation of the procedure or in real-time. However, it is yet unclear whether impacts of such therapeutic stimulation paradigms are also reflected indirectly in other downstream modular networks.

Here we present the outcome of a consortium under the European Union Seventh Framework Program (CORONET), targeted at developing a biohybrid interface between biological and artificial neural networks. We describe the designed setup and show how it can integrate the activities of neural networks *in vitro* with biomimetic hardware networks.

The hardware components are a CMOS-based VLSI, denoted the NeuroSoC system. These components may be integrated, functionally and physically, with neural systems. The overall NeuroSoC system, is built of nine individual NeuroSoCs and a support system realized on a commercial Field Programmable Gate Array (FPGA). This design allows routing of action potentials and communicating with both a host PC and the neural biological network. The NeuroSoC implements biophysical short-term dynamics and a large network size (320 neurons and up to 1.4 million presynaptic spike inputs per chip), for a realistic counterpart to the biological network. Output spikes of the neuron influence both its spike frequency adaptation (SFA) as well as its downstream presynaptic short-term plasticity (STP). The biological neural network is connected in a closed-loop to the NeuroSoC system via Ethernet, where the host PC is running a Matlab Simulink program which enables real-time configuration, monitoring and control of the experiment, with a sub-milliseconds delay.

We couple the hardware network to a biological network, comprises a large-scale, random network of cortical neurons *in vitro*. These networks develop from a culture of dissociated neurons, which form functional connections via synapses. It has been shown that such networks maintain properties of cortical networks *in vivo*, such as the cell type distribution and the response dynamics (Marom and Shahaf, 2002). When embedded on Micro-Electrode Arrays (MEAs), this experimental setup enables stimulation and recording from tens to hundreds of neurons simultaneously, at high spatio-temporal resolution (Gross et al., 1977, 1993; Jimbo et al., 1998; Massobrio et al., 2015). Therefore, it has been a very useful experimental model for neural connectivity, cellular and synaptic physiology, learning and synchronization (Shahaf and Marom, 2001; Chiappalone et al., 2008; Shahaf et al., 2008; Gal et al., 2010; Le Feber et al., 2010; Wallach et al., 2011; Kaufman et al., 2014; Keren and Marom, 2014, 2016; Reinartz et al., 2014; Haroush and Marom, 2015).

The coupling is configured by feeding the output of the 60 MEA channels, following spike detection, to a predefined subset of all 2880 neurons of the NeuroSoC network. The connection is made via Ethernet and the host PC enables the configuration, live experiment monitoring and control. The strength of recurrent and background connections is finely balanced for the hardware network to experience short population spikes.

Such neural networks *in vitro* have been already integrated with electronic devices, in order to record and stimulate activity (Wagenaar et al., 2005; Bontorin et al., 2007; Rolston et al., 2010; Keren and Marom, 2014). Moreover, progress in very-large-scale integration (VLSI) has advanced the design of complex integrated circuits and system-on-chip (SoC) devices (Greenwald et al., 2016). Bidirectional neural interfaces have become a focus for investigation, where stimulation and PID control parameters are explored (Liu et al., 2017), and advancements are also made in the coupling to *in vitro* networks (Chou et al., 2015).

Here we describe an advanced hybridization setup – where the integrated hardware device is an artificial neural network demonstrating complex architecture and response dynamics, and the coupling is with short, millisecond range, processing, and stimulation delays.

We first present the feasibility to functionally couple the biomimetic hardware network with an *in vitro* neural network. Then, we demonstrate the tightness of the coupling by implementing a closed-loop control circuit – where the hardware network activity is modified via an indirect stimulation, which is provided to the biological network. We show also that modifying the activity of the biological network, affects functional properties of the hardware NeuroSoC network. These examples raise questions regarding possible impacts of local stimulation on other networks in modular constructs. Whether such long-range effects of stimulation are a desirable target or not, they are important to study when developing neural control interfaces.

## Results

For coupling the *in vitro* networks with the biomimetic network, it was configured on all 2880 neurons of the NeuroSoC system (Figure 3A). Specifically, we can obtain bursting behavior as observed in cultured networks (Shahaf and Marom, 2001) (see Figure 3B for obtainable behavior range). We use the bursting behavior seen in graph 2 of Figure 3B as viable counterpart to the *in vitro* network.

The feasibility to couple the hardware network with the biological culture, is demonstrated first by showing a congruent synchronization between the two networks, when transferring all biological spiking activity in real-time as inputs to the hardware network. We show that due to the tight coupling, it is also feasible to implement a control circuit which reads the activity of one network while providing stimulation to the other (hence, considers the two as a unified compartment). This design creates a bidirectional interaction between the networks – while the hardware network receives as inputs the spiking activity from the biological network, also the biological activity is stimulated in an intensity which represents the activity level of the hardware network (as illustrated in Figure 4A). The circuit is comprised of the following steps: Sampling the activity of the hardware by a subset of 60 neurons, similarly, to the recording of the culture activity; This activity is then sent to a PI controller algorithm, which calculates the appropriate stimulation amplitude to be applied next; This stimulation is provided to the biological network. The transmission of data packets from culture to hardware and back spans the order of 1 ms. The control parameter is the probability of a network synchronization event to occur following a stimulation. This measure reflects the general activity level, as evoked activity is dominating the network under such stimulation frequency. The control target value of probability was of a sin shape (inset of Figure 4B). Both networks followed this target [see also Wallach et al. (2011) and Keren and Marom (2014) for further technical details of the control algorithm]. The control efficiently modified the activity level of both networks to the target pattern, while eliminating the typical random fluctuations (even though in fact no direct stimulation is provided to the hardware network. See Figure 4C).

Next, we explore how alterations in activity levels in the biological network are reflected in the hardware network. Under this design, the biological network activity level is controlled to maintain two different activity levels, each for more than an hour, while being coupled to the hardware network. This is shown in Figure 5A by three such coupling experiments. Due to the coupling, both the biological and the hardware networks are, similarly, changing between high and low activity levels (Figure 5A, depicted blue and black, respectively). To modify the activity level of the biological network, a similar control algorithm is used to calculate the stimulation amplitude required to either increase or decrease the activity level (Figure 5B). The hardware network activity, however, is not exposed directly to stimulation, hence, any changes are evoked by the activity of the biological network (Figure 5C). Nevertheless, the hardware network activity is being efficiently controlled indirectly via stimulating the biological network.

Moreover, while the activity level of both networks is being congruently modified, we find that additional characteristics are affected as well on both sides. Propagation becomes faster during high activity level. Propagation speed in this context is derived from propagation delays, calculated as the inter-spike-intervals during the recruitment rate of an evoked synchronization. Figure 6A demonstrates that this effect on propagation times, occurs congruently in both networks of the biohybrid. Congruently, synchronization latencies are also altered during high activity level, becoming shorter (inset to Figure 6A). We also find that network synchronization duration is altered between high and low activity levels, in a similar direction in both biological and hardware networks. From a spatial perspective, we find that maintaining the activity level at a higher or lower level, results in an exploitation of different propagation paths across the biological network (Figure 6B, left, a pair-wise similarity matrix, between all response recruitment orders. Note two distinguished groups of responses). This is reflected to some extent in the hardware network as well (Figure 6B, right panel). Of all features, it seems that propagation paths are more affected by the intensity of stimulation, as this feature is mainly altered in the biological network which received the stimulation intensity directly. Therefore, this result suggests that while it can be possible to modify propagation paths using stimulation intensity, it might not be as efficiently modified in another downstream network.

All the described examples for coupling experiments (each comprised of three experiments), were set to a connectivity strength of 20%, but were then replicated with a connectivity of 10% without affecting the results.

## Discussion

We present a setup for coupling a biological neural network with a neuromorphic hardware network. This experimental setting enables to design a real-time functional communication between neural networks, which is well-monitored from one hand while allowing access to complex features of neural networks from the other.

The bidirectional coupling between the networks is implemented by: transferring in real-time the biological spiking activity as inputs to the hardware nodes; modifying the stimulation input provided to the biological network according to the hardware activity. Here, the biological spiking parameters being transferred to the hardware are spike time and electrode identity. The hardware activity is affecting the stimulation of the biological network, by changing its stimulation amplitude. However, this setup allows simple modification of this experimental design and the characteristics of the coupling. For example, the feedback from the hardware to the biological network can be implemented using other stimulation parameters, as stimulation frequency, the spatial pattern of stimulation, etc.

There are various possibilities for implementing such interfaces, which are being currently investigated. These designs can include an *in vivo* or an *in vitro* biological network, can use intracellular or extracellular recording electrodes, can be implemented in a hardware or a software level, can use discrete components or integrated circuits (IC), and digital or analog data processing (Reger et al., 2000; Jung et al., 2001; Masson et al., 2002; Carmena et al., 2003; Nowotny et al., 2003; Oprisan et al., 2004; Whittington et al., 2005; Potter et al., 2006; Bontorin et al., 2007; Novellino et al., 2007). Here, the hardware NeuroSoC network is a SC circuit, which exhibits significantly more robust and reproducible behavior than conventional subthreshold neuromorphic circuits. Even though SC circuits have been typically used with simple membrane leakage current and synaptic transmission (Vogelstein et al., 2007; Folowosele et al., 2009), we implement a more biologically realistic model with conductance-based synapse types and spike-frequency adaptation (Noack et al., 2014, 2015).

We show that using this implementation we can generate a similar pattern and dynamics of activity in the two networks. Such tight coupling between the networks is achieved also under sparse connectivity (tested as low as 10% of connection probability). Moreover, during this coupling, various activity features are also altered congruently between the coupled networks. In both networks, propagation of evoked synchronies is faster when activity is pushed to higher levels by a closed-loop control stimulation. This potentially reflects increased excitatory resources when activity is pushed to higher rates (Haroush and Marom, 2015). Both networks also show shorter latencies to synchronization during higher activity rates. This might suggest that even though the stimulation altering the activity rate is local, it has the potential to alter activity features also of other sparsely connected networks. This is an example of how this experimental design can be relevant to questions of impacts of stimulation on neural functional properties, when embedded in a modular organization (for evidence of such impacts in single networks, see Jimbo et al., 1998, 1999; Madhavan et al., 2006; Chao et al., 2007; Chiappalone et al., 2008; Vajda et al., 2008; Bologna et al., 2010; Le Feber et al., 2010; Keren and Marom, 2014). Interestingly, changes in stimulation amplitude and in response probability, did not affect consistently the network response amplitude. This could be related to more heterogenous neural latencies under higher stimulation amplitudes (Keren and Marom, 2014): if neurons reach their firing peak at different times, there would not be a consistent change in the overall synchrony amplitude. This suggests that the changes in hardware response probability are not elicited by differences in the overall biological network response, but by individual neurons properties during the recruitment time. The biological network is being controlled by the intensity of stimulation which causes faster/more spread direct responses, and the hardware network is then, respectively, controlled by these individual responses properties (propagation delays, firing rate and individual neurons latency changes).

Overall, we present an experimental setup for studying neural-neuromorphic coupling dynamics and impacts of stimulation on such modular constructs. This setup enables to integrate the activities of an *in vitro* MEA setup with a hardware network, in real-time, with a short time delay. Specifically, such experimental setup provides the feasibility to explore many important questions, from signal propagation in modular constructs, functional transference between networks, impacts of the density of modular connections, local versus global stimulation effects to external intervention in activity of modular constructs. Hopefully, the efficient experimental access to these questions, would enable to expose significant considerations for developing brain-neuromorphic interfaces.

## Materials and Methods

### Hardware System

**FIGURE 1:**
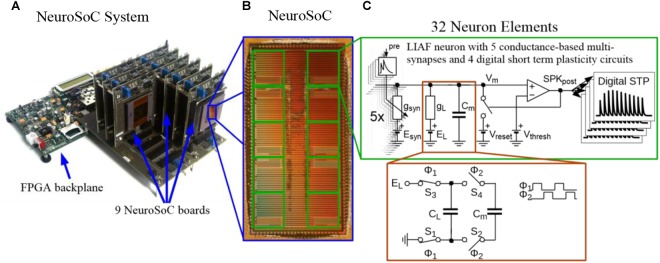
The Neuromorphic System-on-Chip (NeuroSoC) **(A)** The overall NeuroSoC system, with nine individual NeuroSoCs in combined operation; a support system realized on a commercial Field Programmable Gate Array (FPGA) supplies action potential routing and configuration between the individual NeuroSoC chips; Also, the FPGA backplane enables communication to the host PC and the neural culture. **(B)** The NeuroSoC implements biophysical short-term dynamics and a big network size (320 neurons and up to 1.4 million presynaptic spike inputs per chip) for a realistic counterpart to the neural culture. **(C)** Circuit of one of the 10 neuron groups contained on the NeuroSoC, each with 32 neurons realized in switched capacitor technique (see red insert), each with five types of conductance based synaptic input.

**FIGURE 2:**
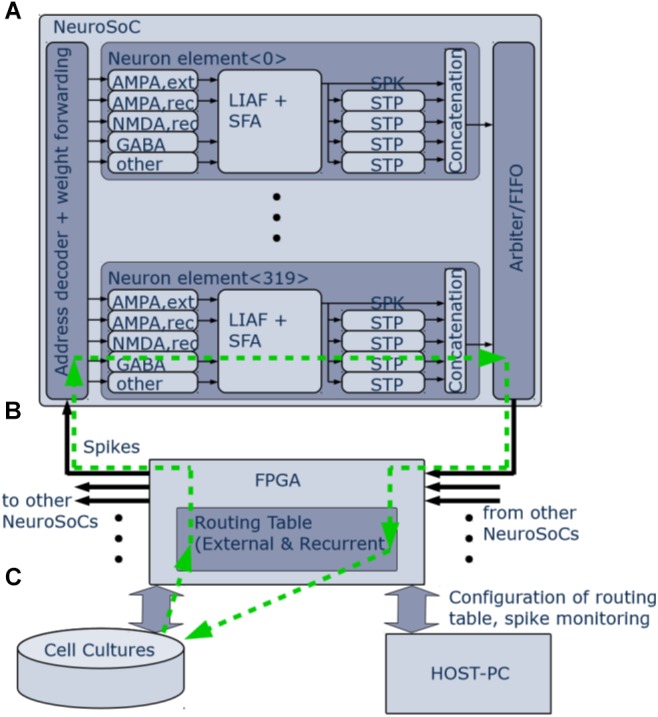
Logical organization of the NeuroSoC system. **(A)** Single NeuroSoC and its processing elements with sample spike processing chain illustrated in the green, dashed line. A spike enters via the address decoder and gets transmitted to the respective multi-synapse; this in turn generates a conductance change (excitatory or inhibitory) on the SC-neuron. Output spikes of the neuron influence its spike frequency adaptation (SFA) as well as its downstream presynaptic short-term plasticity (STP). The spike address plus its four STP values are concatenated into a pulse packet and sent off-chip. **(B)** The FPGA backplane contains playback memory for background stimulation and the routing table between the NeuroSoCs; as well, it enables communication with the neural culture setup and the controlling host PC. **(C)** Neural cultures are connected in a closed loop to the NeuroSoC system via Ethernet; the host PC enables configuration, live experiment monitoring and control.

While we do not give a fully detailed description of the hardware circuits, we do describe to some extent the hardware part of the system in the following section. A more detailed overview of our Neuromorphic System-on-Chip (NeuroSoC, Figure 1B) can be found in Noack et al. (2014). As the NeuroSoC should only replicate short-term dynamics, it omits the usual synaptic matrix with its long-term plasticity computation (Qiao et al., 2015). Thus, instead of the conventional neuromorphic chip layout with presynaptic adaptation at the input, a synaptic matrix and postsynaptic neurons, the NeuroSoC can be thought of as a collection of neuron building blocks, the neuron elements (see right half of Figure 1, respectively, Figure 2A for an organizational overview). The neuron elements are grouped in 10 groups of each 32 neuron elements (for a total of 320 neuron elements per chip). Overall, the system in Figure 1A contains 9 NeuroSoCs with 2880 neurons, 14400 conductance-based multi-synapses (equivalent to 12.7 M synapses) and 11520 presynaptic STP circuits. Analog biasing voltages for neurons and synapses (e.g., synaptic reversal potentials, membrane resting potentials, etc.) are generated via digital-analog converters on chip.

Figure 2 gives an organizational overview of the hardware system. The incoming synapses are conductance-based. Five individually configurable multi-synapses per neuron allow to model five separate synapse characteristics of either AMPA, NMDA, or GABA type. One inhibitory conductance is triggered by the neuron itself and thus acts as spike-frequency-adaptation. The output of the neuron feeds into four digital short-term adaptation modules. Each of these can be configured individually and thus provide the neuron with different types of synaptic adaptation for its downstream connections. All pulses are routed via an FPGA (Figure 2B) that also handles the routing in between the nine NeuroSoC chips that make up the system. Routing was designed to allow for arbitrary network topologies. Each of the four STP outputs of every neuron has 3.4 k routing entries in the FPGA routing table, which can either target individual multi-synapses of neurons inside the system or addresses outside, the latter being used for communication with other devices such as the neural cultures setup. Each routing entry includes a 6-bit synaptic weight, which is sent to the NeuroSoC together with the adaptation state of the source STP circuit. Both values are multiplied on-chip giving the resulting synaptic efficacy value to be added to the target multi-synapse. The NeuroSoC receives the incoming FPGA pulses via an address decoder, while an arbiter handles conflicts between outgoing pulses. The FPGA also handles the host communication and control (Figure 2C). The green arrows illustrate a sample spike path from/to the culture. The Ethernet-based interface to the neural cultures is also implemented on the FPGA (George et al., 2015). For simplicity, the clock for the SC circuits is supplied by the FPGA but could in principle also be generated on chip (Eisenreich et al., 2009).

### Hardware Components

The neurons and synapses on the NeuroSoC are carried out in the Switched Capacitor (SC) circuit technique. This means that conductances are determined by the switching frequency in connection with the capacitance (see Figure 1C). This reliance on frequencies and capacitance ratios, instead of process-dependent transistor parameters, lets the NeuroSoC exhibit very robust and reproducible behavior, ideal for biological experiments. A specialized switching circuit has been used to reach the necessary timescales in the order of 1 s (Mayr et al., 2016), which are otherwise hard to obtain in semiconductor circuits due to leakage currents (Roy et al., 2003). Figure 1C shows the analog circuits comprising the neuron element. In essence, this is a differential SC realization of a Leaky Integrate-and-Fire neuron with spike-frequency-adaptation and conductance-based multi-synapses at the input. The NMDA synapses constitute a special case as they exhibit a non-linear dependence on the membrane voltage plus a saturation. We have approximated this by an analog computation along a piecewise linear curve (Noack et al., 2014). The biological realism we aimed for in our SC circuits compares favorably with the literature, i.e., SC circuits were previously only used for simple membrane leakage current generation and synaptic transmission (Vogelstein et al., 2007; Folowosele et al., 2009; Noack et al., 2015).

When modeling the synaptic conductance in SC technique, a fixed frequency would result in a fixed conductance. Therefore, a digital circuit generates an exponentially-decaying frequency to emulate an instantaneous-onset, exponentially-decaying synaptic conductance. Each of the five synaptic conductances is implemented as a multi-synapse, meaning that multiple synaptic connections are modeled by overlaying them linearly on the same synaptic circuit. Hence the instantaneous increase will be re-triggered for each synaptic input on that conductance type and subsequent exponential decay performed on the accumulated value. Due to its widely configurable behavior range, we employ a model of presynaptic STP in the NeuroSoC (Markram et al., 1998). A fully digital translation of the model is used, as it was comparable in power and area to an SC implementation and offered superior programmability and repeatability. It transmits its 6-bit adaptation state off-chip for further routing in the FPGA. This state is used at the input of the next NeuroSoC as a dynamic synaptic weight value. In the same spirit as the multi-synapses, we did not implement a specific presynaptic adaptation circuit per single synapse configured in the system. Instead, four different realizations of the STP circuit driven by the same neuron provide for differently-configured flavors of presynaptic adaptation to postsynaptic neurons. That is, one STP channel could be configured for facilitation, one for depression, one for combined facilitation and depression (Noack et al., 2011) and each postsynaptic neuron can be configured in the FPGA routing table to receive one of those flavors. Please note that the multi-synapses do not implement long term plasticity, as this is usually a monosynaptic process, i.e., it would need individually realized synapses. This was intentionally omitted as we are only replicating short-term effects of the biological network. Due to the corresponding savings in silicon area, this in turn allowed us to realize more neurons per chip and it also allowed potentially very densely connected networks via the multi-input synapses. However, we could still implement long-term plasticity on the permanent weights via a plasticity processor on the FPGA similar to Friedmann et al. (2013) and George et al. (2015), as the FPGA has access to the weights as well as to, e.g., the pre- and postsynaptic spike trains.

### Biomimetic Network

**FIGURE 3:**
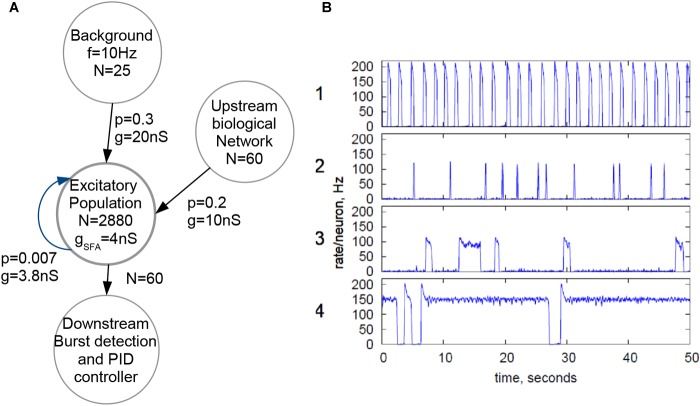
Configuration of the NeuroSoC system. **(A)** Hardware network structure, with background stimulus provided by the FPGA and a single excitatory population configured with all 2880 neurons available; spike activity from the culture is added as additional synapses into a fraction of the excitatory network. Each circle denotes a population of neurons/spike sources (e.g., the Poisson background provided by the FPGA at the top). N denotes the number of neurons/spike sources in said population, with the arrows indicating linkages and synapse direction; p is the connection probability between each pair of pre- and postsynaptic neuron of the two populations and g the conductance of the synapse. **(B)** Four sample population behaviors (population average firing rate, normalized to single neuron); the parameters of the excitatory network and the background stimulus can be configured for a range of behaviors; for similarity with the neural culture behavior, behavior type 2 was chosen, resulting in the parameters given in **A**.

Figure 3A shows the topology employed for the biohybrid experiment on the NeuroSoC system. At its center, all 2880 neurons of the system (distributed across the nine NeuroSoCs) are configured as an excitatory recurrent network with SFA. This network size approximately resembles the number of neurons present in the region of the MEA. For parametrization of the network, we use a theory-guided approach (Giulioni et al., 2012; Partzsch et al., 2019), employing mean-field theory. While smaller neuromorphic systems have been employed for a mean-field approach (Giulioni et al., 2012), the underlying diffusion approximation asks for synaptic fan-in being significantly smaller than the network size, which is a second motivation for fully utilizing the hardware system. In turn, recurrent connection probability was set to a low value of 0.007, i.e., on average a single neuron is recurrently connected to 20 other neurons. Feeding into this network is a background stimulus of 25 Poisson spike sources at 10 Hz, with each spike source connected to the recurrent network with *p* = 0.3 and a maximum synaptic conductance of *g* = 20 nS. Also feeding into this network is the output of the spike detection operating on the 60 MEA channels, i.e., 60 channels of biological spikes. Aided by the good statistical adherence of our implementation to the model behavior, we can configure the recurrent network for a wide range of dynamical regimes, guided by mean-field theory (Giulioni et al., 2012; Partzsch et al., 2019), see Figure 3B. For the hybrid experiments in this paper, the SFA strength, strength of recurrent connections and the background are finely balanced for the network to experience short population spikes, as shown in behavior 2 of Figure 3B. Parameters of this configuration are given in Figure 3A. In this setting, the network can have spontaneous population spikes driven by the background stimulus as well as evoked spikes driven by the input from the neural cultures.

**FIGURE 4:**
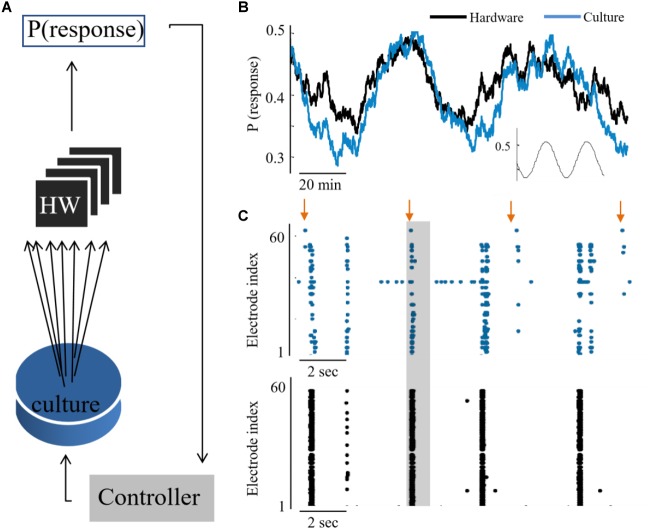
Hybrid coupling and control circuits. **(A)** Scheme of the coupling and control between the culture and neuromorphic device: a PI controller receives the activity of the hardware network, calculates the required stimulation amplitude to reach a predefined activity level in hardware, but then this stimulation is applied to the biological network. **(B)** The output of synchronization probability of an example experiment, where a culture and hardware networks are coupled (see methods for the control algorithm). **(C)** Extracts of aligned activity as recorded in the biological network (blue) and hardware (black). Each point depicts a single spike detected in one of the electrodes (indexed in the vertical axis). Arrows depict stimulation times and shaded regions depict the time window for detection of an evoked burst (10–800 ms after stimulation).

**FIGURE 5:**
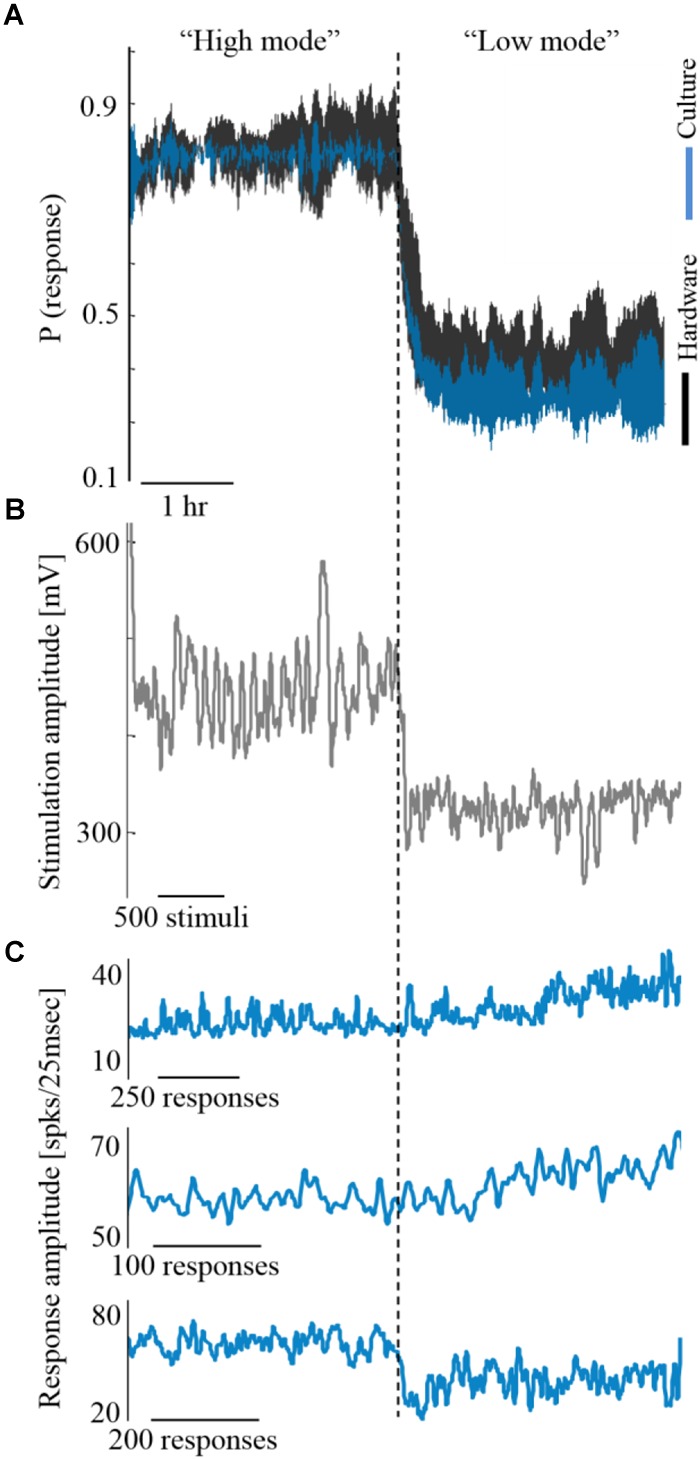
Coupling during different activity levels. **(A)** Three experiments exemplifying coupling while the biological network is controlled to high and low activity levels (blue). Trace margins are standard deviation values across the three cultures. Hardware network activity is depicted black. **(B)** The stimulation amplitudes required for controlling one of the biological networks, as computed by the control algorithm. Higher stimulation amplitudes are required for maintaining higher activity level (shown for one of the cultures, although all three experiments showed a similar trend). **(C)** The evoked synchronization sizes of the three cultures show no consistent relation to the activity level, hence this is not the feature that modifies the hardware network activity.

**FIGURE 6:**
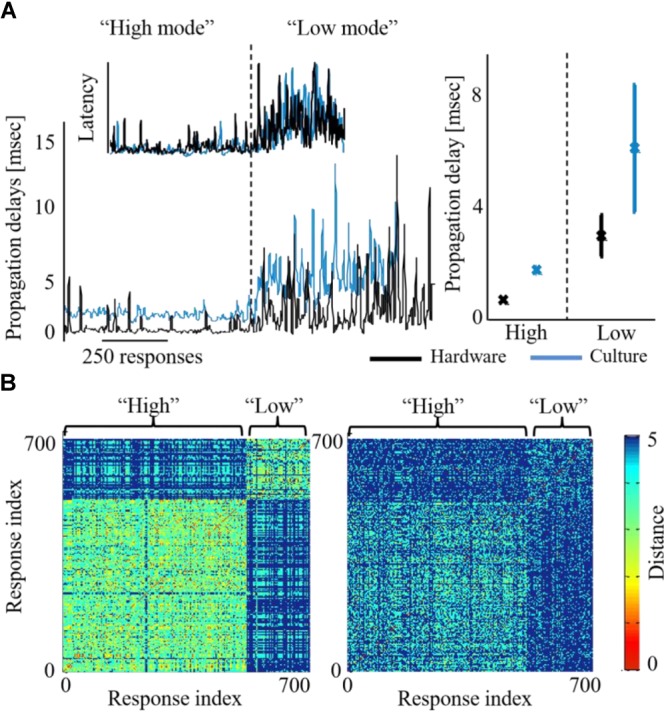
Activity features modified across both biohybrid networks. **(A)** Propagation delays of synchronizations, under either high or low activity leve l (blue depicts the biological network and black the hardware one). Inset shows the respective alteration of synchronization latency, calculated as the time from stimulation to the synchronization peak. Right panel: average latency values across three coupling experiments, error bars show standard deviation (*p* < 0.05 in a *t*-test between the activity levels for both biological and hardware networks). **(B)** Synchronization recruitment order in the biological (left) and the hardware (right) networks, during high and low activity levels, of a single coupling experiment. Pair-wise difference between propagation paths are presented (calculated with the Levenshtein distance metric), for the first five responding electrodes, across all evoked bursts consecutively [see Shahaf et al. (2008) for details]. The distance between order vectors of evoked burst *i* and *j* is color coded in pixel (*i*,*j*) between red (0, identical order) and blue (5, no identity in order). Different propagation paths between the high and the low activity levels, would result in a non-homogenous image with two visible squares (where the within-level comparison square has a warmer shade, i.e., lower differences).

### Cell Preparation

Cortical neurons were obtained from newborn rats (Sprague-Dawley) within 24 h after birth using mechanical and enzymatic procedures described in earlier studies (Marom and Shahaf, 2002). Rats were anesthetized by CO_2_ inhalation according to protocols approved by the Technion’s ethics committee. The neurons were plated directly onto a substrate-integrated multi electrode array and allowed to develop into functionally and structurally mature networks over a period of 2–3 weeks. The number of plated neurons was of the order of 450,000, covering an area of about 380 mm^2^. The preparations were bathed in MEM supplemented with heat-inactivated horse serum (5%), glutamine (0.5 mM), glucose (20 mM), and gentamycin (10 μg/ml), and maintained in an atmosphere of 37°C, 5% CO_2_ and 95% air, also during electrophysiological recording.

### Electrophysiology

An array of Ti/Au extracellular electrodes, 30 μm in diameter, spaced 500 μm from each other and located in the center, was used (MultiChannelSystems, Reutlingen, Germany). A commercial amplifier (MEA-1060-inv-BC, MCS, Reutlingen, Germany) with frequency limits of 150–3,000 Hz and a gain of ×1024 was obtaining data. Data was digitized using data acquisition board (PD2-MF-64-3M/12H, UEI, Walpole, MA, United States). Each channel was sampled at a frequency of 16 kHz. The sampling rate was defined to allow multiple samples of the extracellular action potential, especially during the most rapid voltage change phase. In an intracellular action potential, the steepest voltage change lasts for about 200 μs, during the depolarization. Therefore, the highest frequencies during an extra cellular action potential are in this frequency range (Gydikov and Trayanova, 1986; Mitra, 2007). The insulation layer (silicon nitride) was pre-treated with polyethyleneimine (Sigma, 0.01% in 0.1 M Borate buffer solution). Data acquired was analyzed using Matlab (Mathworks, Natick, MA, United States). Specifically, Simulink was used to process, analyze and transfer data on-line. Voltage stimulation was applied in a monophasic 200 μs square pulse 100–1000 mV, through extracellular electrodes, using a dedicated stimulus generator (MCS, Reutlingen, Germany). We based the choice of these parameters on previous works which focused on studying these aspects. These stimulation parameters were shown to generate efficiently action potentials in single neurons, for long time scales.

### Analyses

**FIGURE 7:**
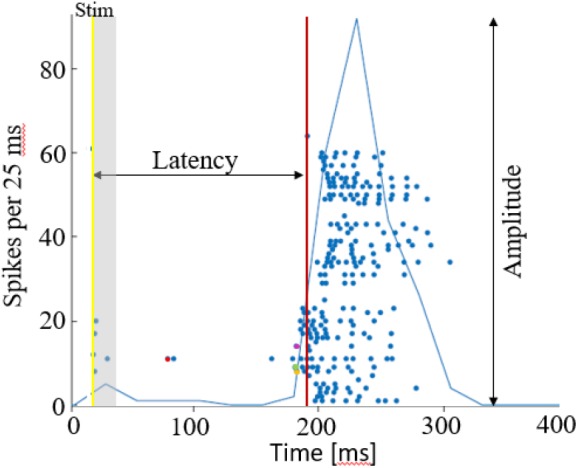
Network synchronization parameters. A raster plot of a single network burst, where each dot depicts an action potential by the time and electrode index. The latency is calculated as the time from stimulation time (yellow line), to the synchronization detection (red line). The amplitude of synchronization is the maximal sum of action potentials, in 25 ms. Action potentials occurring within the first 15 ms after stimulation are excluded from the propagation path analysis, assuming these are caused by direct excitation from the stimulation current. The propagation path is then identified by the order of responding electrodes, in this case it the first 4 are electrodes 11 (red), 9 (green), 8 (yellow), 14 (purple). The propagation speed/delay, would be in this case the time interval between these first spikes occurring in each of the electrodes.

Action potentials were detected by threshold crossing which is defined separately for each of the recording channels at the beginning of an experiment (6 × standard deviation of a 2 s voltage trace). A refractory period of 6 ms was considered. The detected activity in the electrodes can originate from one to two neurons. Nonetheless, it has been shown that using this spike detection method maintains the functional phenomena observed when using spike sorting (Eytan and Marom, 2006; Shahaf et al., 2008). Therefore, we found that for the purpose of this study it is sufficient if activity is comprised of 1–2 different neurons, which we consider here as an activation unit. Spike sorting could be added to the algorithm if individual spike shape properties are studied, for example. Detection of network bursts (i.e., synchronization events) was performed on-line by threshold crossing of the summed action potentials, binned to 25 ms. Exact threshold was determined according to 25% of the active electrodes, typically 20 action potentials. See Figure 7 below for illustration of a network synchronization parameters: each dot indicates a single action potential, described by its time stamp (*x*-axis) and the recording electrode index (*y*-axis). The solid blue line represents the sum of action potentials. The figure indicates the electrical stimulation time (yellow vertical line), the burst detection time (red vertical line), latency from stimulation to occurrence of synchronization, the amplitude of synchronization event and the propagation order.

### PI Algorithm

A *Proportional-Integral-Derivative (PID)* controller was realized on the xPC target system (Levine, 1996). The input to the controller is the error signal,

(1) $$e_{\text{n}}=P_{\text{n}}^{*}-{\overset{⌣}{P}}_{\text{n}}$$

where *P*^∗^_n_ and ${\overset{⌣}{P}}_{n}$ are the desired and estimated response probabilities at the *n*th stimulus, respectively. The output of the controller is generally composed of four components,

(2) $$A_{\text{n}}=A_{\text{baseline}}+g_{\text{P}}e_{\text{n}}+g_{\text{P}}e_{\text{n}}+g_{\text{I}}\sum_{\text{i}=1}^{n}e_{\text{i}}+g_{\text{D}}(e_{\text{n}}-e_{\text{n}-1})$$

where g_P_, g_I_, and g_D_ are the proportional, integral, and derivative gain parameters, respectively (typically g_P_ is 1, 400 mV; g_P_*e*_n_ + *g*_I_ is 0.2, 80 mV; and g_D_ is 0), and A_baseline_ is the baseline amplitude bias (set to the amplitude which evokes the desired response probability in an open loop stimulation prior to the experiment). In this setup the stimulation amplitude range is between 100 and 1150 mV. Reaching the limit of this stimulation amplitude range, results in a saturation of the input signal to a constant uncontrolled signal, while the integrative error value steadily increases. Hence, for maintaining the control, stimulation within this range is required.

### On-Line Estimation of Network Response Probability

The control algorithm uses a reduced binary response characteristic, representing the occurrence of a synchronized network response. Let *s*_i_ be an indicator function, so that *s*_i_ = 1 if the network generated a network burst within a predefined interval after the *i*th stimulus and *s*_i_ = 0 otherwise. The time interval for detecting an evoked synchronization event following a stimulation was set to 10–800 s. This interval was chosen according to previous studies which validated this time as the optimal for separating evoked synchronization from a spontaneous one (Gigante et al., 2015; Bauermeister et al., 2018). We denote as ${\overset{⌣}{P}}_{n}$ the estimated probability, calculated at time *t > t*_n_, based on the set of responses {*s*_1_,*s*_2_,…, *s*_n_} to stimuli given at times {*t*_1_,*t*_2_,…, *t*_n_}. A weighted average was realized by using the recursive formula,

(3) $${\tilde{P}}_{\text{n}}=(1-e^{-\frac{t_{\text{n}}-t_{\text{n}-1}}{\text{τ}}})\cdot s_{\text{n}}+e^{-\frac{t_{\text{n}}-t_{\text{n}-1}}{\text{τ}}}\cdot {\tilde{P}}_{\text{n}-1}$$

the estimation time-constant, τ, was typically set to 250 s.

## Ethics Statement

This study was carried out in accordance with protocols approved by the Technion’s ethics committee.

## Author Contributions

HK and SM set up the *in vitro* component of the biohybrid. JP and CM set up the hardware component of the biohybrid. HK led the biohybrid measurements and data analysis, with JP and CM contributing. All authors contributed to the writing of the manuscript and approved it for publication.

## Conflict of Interest Statement

The authors declare that the research was conducted in the absence of any commercial or financial relationships that could be construed as a potential conflict of interest.
